# Comparison of the conventional Henry approach and trans-flexor carpi radialis approach for the treatment of distal radius fracture: A retrospective cohort study

**DOI:** 10.1097/MD.0000000000031936

**Published:** 2022-12-09

**Authors:** Bum-Jin Shim, Do-Young Kim, Sang-Soo Lee, Min-Soo Cho, Jung-Taek Hwang

**Affiliations:** a Department of Orthopedic Surgery, Kyungpook National University Chilgok Hospital, Buk-gu, Daegu, Republic of Korea; b Department of Orthopedic Surgery, Chuncheon Sacred Heart Hospital, Hallym University Medical College, Chuncheon-si, Gangwon-do, Republic of Korea.

**Keywords:** distal radius, flexor carpi radialis approach, fracture, henry approach

## Abstract

Few studies have compared the clinical outcomes of the conventional Henry approach and trans-flexor carpi radialis (FCR) approach. The purpose of this study was to compare the clinical and radiologic outcomes of the conventional Henry approach and trans-FCR approach for the treatment of distal radius fractures. We compared 20 wrists that underwent the conventional Henry approach with 20 wrists that underwent the trans-FCR approach for open reduction and internal fixation of distal radius fracture. The clinical and radiological parameters were checked at 3 months, 6 months, and 1 year after surgery. A visual analogue scale score, the modified Mayo wrist score, range of motion, and grip strength were collected. In addition, tenderness in the area of the FCR tendon were assessed. Regarding radiologic evaluations, the radial inclination, radial height, volar tilt, and ulna variance were measured. In the trans-FCR approach group, 15 patients complained of tenderness in the area of the FCR tendon at 3 months after surgery, which was significantly higher than those of conventional Henry approach group (*P* < .05). In the conventional Henry approach group, the tenderness had resolved spontaneously by 1 year after surgery in 19 patients. The trans-FCR approach can cause discomfort such as tenderness to the area of the FCR tendon compared to the conventional Henry approach, but there is no significant difference in the final clinical and radiologic outcomes.

## 1. Introduction

Distal radius fractures are one of the most common upper extremity fractures in adults.^[1,2]^ Recently, the use of volar locking plates for the surgical treatment of distal radius fractures has increased. Even though there is debate on the preferred approach, the most common surgical approaches are the conventional Henry approach and modified Henry approach, also called the trans-flexor carpi radialis (FCR) approach.^[3]^

The 2 approaches differ in the superficial interval used to expose the deep volar compartment. The conventional Henry approach requires identification and protection of the radial artery and ulnar mobilization. This approach has the advantage of preserving the FCR tendon sheath and the palmar cutaneous branch of the median nerve. The trans-FCR approach proceeds deep into the forearm fascia via the FCR tendon sheath and allows ulnarward retraction of the FCR tendon. Although this approach can avoid the risk of dissection and injury to the radial artery, the palmar cutaneous branch of the median nerve can be injured during surgery. This approach often requires elevation of the flexor pollicis longus (FPL) tendon and postoperative FPL weakness may occur.^[4]^ Additionally, since the tendon sheath is incised, there is a possibility of adhesions.^[5–7]^

Although several studies have reported the clinical outcomes of various surgical approaches for distal radius fracture, few studies have compared the clinical outcomes of the conventional Henry approach and trans-FCR approach.^[8–11]^ Therefore, the purpose of this study was to compare these 2 surgical approaches for the treatment of distal radius fracture and to evaluate which approach provided better outcomes. We hypothesized that incision of the FCR tendon sheath would contribute to an increase in patient discomfort postoperatively.

## 2. Materials and methods

### 2.1. Subjects

Ethical approval for the study was obtained from the local ethics committee (IRB no.: CHUNCHEON 2021-09-002). We retrospectively reviewed all patients who were treated by internal fixation with volar locking anatomical plates for distal radius fractures performed by 1 surgeon (JTH) from March 2012 to September 2013.

The inclusion criteria were as follows: a diagnosis of distal radius fracture, unilateral involvement, and follow-up periods of more than 1 year. The exclusion criteria were as follows: bilateral distal radius fracture, other fractures that might affect ipsilateral or contralateral function of the upper extremities, open fractures, neurovascular injury either preoperative or iatrogenic intraoperative, time to surgery exceeding 2 weeks, and follow-up periods of <1 year.

The indications for surgical treatment included well-established radiological criteria such as a volar or dorsal angulation of 10°, radial inclination angle of 15°, or an intra-articular step of 2 mm, which were analyzed before closed fracture reduction. The radiologic classification was carried out using the Arbeitsgemeinshaft Osteosynthesfragen system, which is identical to the Orthopaedic Trauma Association classification for distal radius fractures. Open reduction and internal fixation were delayed for 5 to 12 days after injury to allow the swollen soft tissue to recover.

Finally, forty consecutive patients were enrolled in this study. The patients were divided into 2 groups according to the surgical approach used. Group 1 consisted of 20 patients who were treated with the conventional Henry approach, and Group 2 consisted of 20 patients treated with the trans-FCR approach. The conventional Henry approach was applied for the first 20 patients from March 2012 to August 2012. Sequentially, the trans-FCR approach was applied for the next 20 patients from September 2012 to September 2013.

### 2.2. Surgical techniques

**Figure 1.**
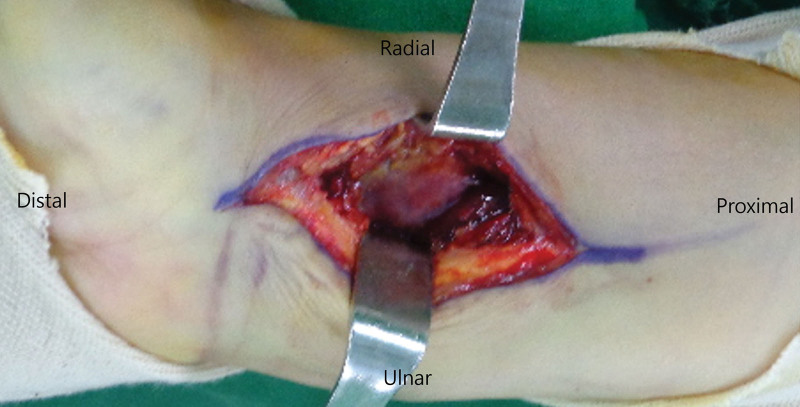
A photograph showing the conventional Henry approach that spare the sheath of the flexor carpi radialis tendon.

In the conventional Henry approach group, a skin incision was made just lateral to the edge of the FCR tendon. The interval of the dissection extended between the FCR and the radial artery to avoid injury to the palmar cutaneous branch of the median nerve, which lies just ulnar to the FCR (Fig. 1). This nerve did not need to be identified if the dissection was maintained to the radial side of the FCR. The difference between the conventional Henry approach and the trans-FCR approach is that the former is performed between the FCR tendon and the radial artery, without opening the FCR tendon sheath, whereas the latter is performed through the FCR tendon sheath.

**Figure 2.**
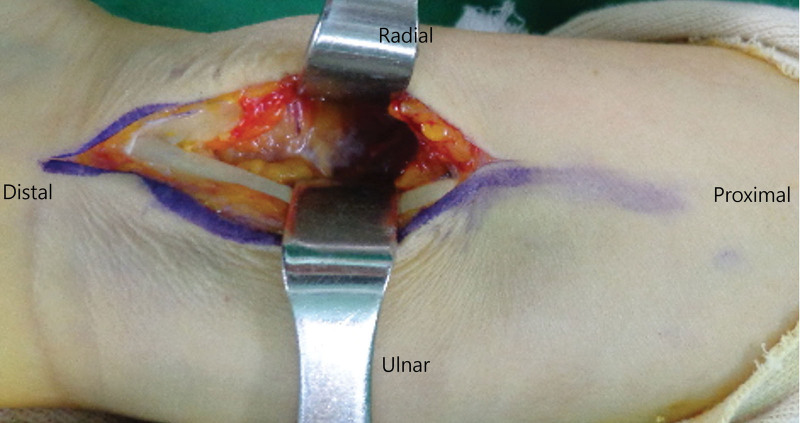
A photograph showing the trans-flexor carpi radialis approach that incise the sheath of the flexor carpi radialis tendon.

In the trans-FCR approach group, a skin incision was made along the FCR tendon, leaving the median nerve protected by soft tissue. The FCR tendon was retracted ulnarly after its tendon sheath was opened (Fig. 2). The FPL muscle was retracted ulnarly, and the pronator quadratus muscle was exposed and incised radially to expose the distal aspect of the radius. The techniques for fracture reduction and application of the locking plate did not differ between the 2 groups. The fracture fragments were mobilized using a combination of sharp dissection, Freer elevators, rongeurs, and distraction as needed and as dictated by the individual fracture pattern. Reduction was achieved with fracture fragment manipulation under fluoroscopic guidance. The reduction was temporarily held using K-wires, if needed, and fixed with a 2.4-mm Variable-Angle LCP® Volar Distal Radius Plate (Synthes, Oberdorf, Switzerland). The specific plate size was determined based on the fracture pattern, anatomical considerations of the patient, and surgeon preference.

### 2.3. Postoperative protocol

Hand therapy, including active finger mobilization, and hand and wrist edema reduction was started immediately after surgery in all patients. All patients underwent volar short arm splint in neutral position for 4 weeks after surgery. Depending on the fracture type and bone quality, active wrist motion was encouraged between 2 and 4 weeks after surgery. Strengthening and weight-bearing exercises were started 6 weeks after surgery. The hardware removal was performed at minimum 1 year after the surgery in all patients.

### 2.4. Clinical and radiographic evaluations

**Figure 3.**
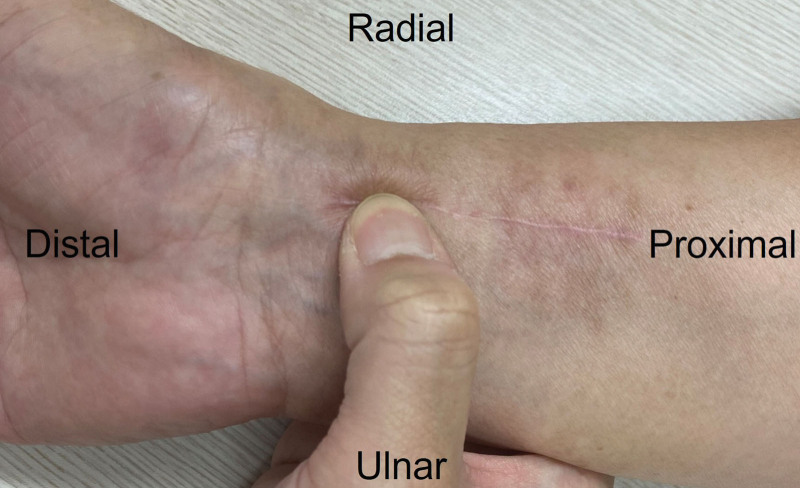
A photograph examining the tenderness and hardness in the area of the flexor carpi radialis tendon.

Clinical and radiological evaluations were performed at 3 months, 6 months, and 1 year after surgery. Regarding the clinical outcomes, a visual analogue scale (VAS) score, the modified Mayo wrist score (MMWS),^[12]^ range of motion (ROM), and grip strength were collected for evaluate the primary outcomes. In addition, for evaluate the secondary outcome, tenderness in the area of the FCR tendon were recorded to judge the degree of adhesion (Fig. 3). Additionally, the presence of complications was evaluated. A 10 point VAS, ranging from 0 (no pain) to 10 (very severe pain), was used to measure pain intensity. The MMWS comprises a total of 100 points which are divided among the evaluator’s assessment of pain (25 points), active flexion/extension arc as a percentage of that on the opposite side (25 points), grip strength as a percentage of that on the opposite side (25 points), and the ability to return to regular employment or activities (25 points). The total score ranges from 0 to 100 points with higher scores indicating a better result. An excellent result was defined as 90 to 100 points, good was 80 to 89, fair was 65 to 79 points, and poor was less than 65 points. Objective results were obtained by measuring the ROM and grip strength using a Jamar dynamometer (Asimow Engineering, Los Angeles, CA). The unaffected, contralateral wrist was examined accordingly and served as an internal control. The scores of tenderness were 0 for not tender, and 1 for tender.

Radiographic evaluations were conducted using standard measurements as described by Kreder et al for radial inclination, radial height, volar tilt, and ulna variance.^[13]^ All radiographic angles were measured and recorded by 2 authors not involved in demographic data acquisition.

### 2.5. Statistical analysis

Power analysis indicated that a total sample size of 40 patients (20 patients in each cohort) would provide a statistical power of 80% with a 2-sided α level of.05 to detect significant differences in the VAS at 1 year after surgery, assuming an effect size of 0.95 (mean difference, 0.85; standard deviation, 0.89). Preoperative and postoperative measurements in each group were compared using Wilcoxon signed-rank tests or paired t test for continuous variables according to the normality of data, and the chi-square test for categorical variables. Additionally, the differences were analyzed using the Mann–Whitney *U* test or *t* test for independent samples according to the normality of data. Statistical significance was set at *P* < .05. Statistical analyses were conducted using IBM SPSS 2019 version 26.0 software (IBM Co., Armonk, NY).

## 3. Results

### 3.1. Patient characteristics

The demographic data of the conventional Henry approach and trans-FCR approach groups are presented in Table 1. There were no remarkable differences in the majority of the variables between the 2 groups. The median age and the median time from injury to surgery were similar in both groups. The median operation time was longer in the trans-FCR approach group (75.00 vs 87.50); however, there was no significant difference (*P* = .100). Additionally, there were no significant differences regarding fracture type between the 2 groups (*P* = .719). There was no follow-up loss of patients.

**Table 1:** Demographic data of the conventional henry approach and trans-flexor carpi radialis approach groups.

Characteristics	Conventional Henry approach (n = 20)	Trans-Flexor Carpi Radialis approach (n = 20)	*P* value
Age (yrs)	62.50 (56.00-69.75)	63.00 (53.25-72.00)	.892
Sex (M:F)	7:13	4:16	.288
Time from injury to surgery (d)	1.00 (0.00-2.00)	1.00 (0.00-1.75)	.470
Operation time (min)	75.00 (66.25-85.00)	87.50 (75.00-95.00)	.100
AO classification			.719
Type A			
A1	0	0	
A2	6	4	
A3	2	2	
Type B			
B1	2	4	
B2	4	7	
B3	4	1	
Type C			
C1	1	1	
C2	0	0	
C3	1	1	

Values in data cells represent median (interquartile range) or number.

AO = Arbeitsgemeinshaft Osteosynthesfragen.

Boldface indicates statistical significance (*P* < .05).

### 3.2. Clinical outcomes

Table 2 shows the clinical outcomes of both groups. Regarding VAS score, it did not show significant difference between the 2 groups at 3 months after surgery (2.0 vs 3.0, *P* = .234). However, when comparing at 6 month and at 1 year after surgery between the 2 groups, VAS score was significantly greater in the trans-FCR approach group (2.50 and 2.00) than in the conventional Henry approach group (1.50 and 1.00) (*P* < .05 and < .01, respectively).

**Table 2:** Comparison of clinical outcomes between 2 groups.

	Conventional Henry approach (n = 20)	Trans-FCR approach (n = 20)	*P* value
VAS			
3 mo	2.00 (2.00–3.00)	3.00 (2.00–4.00)	.234
6 mo	1.50 (0.25–2.00)	2.50 (2.00–3.00)	**.013**
1 yr	1.00 (0.00–1.00)	2.00 (1.00–3.00)	**.004**
MMWS			
3 mo	57.50 (55.00–60.00)	55.00 (55.00–60.00)	.313
6 mo	70.00 (65.00–75.00)	69.25 (60.00–78.75)	.425
1 yr	80.00 (75.00–83.75)	77.50 (70.00–88.75)	.815
ROM (°)			
3 mo	70.00 (61.25–83.75)	70.00 (66.25–75.00)	.956
6 mo	95.00 (85.00–110.00)	90.00 (90.00–98.75)	.521
1 yr	120.00 (111.25–138.75)	112.50 (110.00–123.75)	.099
Grip strength (% contralateral side)			
3 mo	51.00 (46.25–64.00)	47.00 (40.25–55.75)	.110
6 mo	81.50 (75.00–86.25)	79.50 (74.25–84.50)	.516
1 yr	93.00 (91.00–94.00)	94.00 (86.00–101.50)	.765
Tenderness			
3 mo	7 (35.0%)	15 (75.0%)	**.012**
6 mo	3 (15.0%)	8 (40.0%)	.080
1 yr	1 (5.0%)	5 (25.0%)	.080

Values in data cells represent median (interquartile range) or number.

FCR = flexor carpi radialis, MMWS = Modified Mayo Wrist Score, ROM = range of motion, VAS = visual analog scale.

Boldface indicates statistical significance (*P* < .05).

MMWS were found to be similar between the 2 groups in each follow-up examination. The median ROM at 1 year was greater in conventional Henry approach group (120.00 vs 112.50). However, there was no significant difference (*P* = .099). There were no remarkable differences in grip strength at each follow-up between the 2 groups.

In the conventional Henry approach group, the numbers of patients who complained of tenderness in the area of the FCR tendon at 3 months after surgery were 7. In 6 patients, the tenderness had resolved spontaneously by 1 year after surgery without any further intervention or specific treatment. Only 1 patient complained of persisting tenderness at 1 year after surgery.

In the trans-FCR approach group, 15 patients complained of tenderness in the area of the FCR tendon at 3 months after surgery, and there was a significant difference between the 2 groups (*P* < .05). In 10 patients, the tenderness had resolved spontaneously by 1 year after surgery. The follow-up results at 6 months and 1 year were not significantly different between the 2 groups. No complications such as injury of palmar cutaneous branch of median nerve, especially in the trans-FCR approach, and of radial artery, especially in the conventional Henry approach were reported. Also, infection, hematoma, tendon rupture, or complex regional pain syndrome, were not reported.

### 3.3. Radiologic outcomes

According to the radiologic evaluation, no significant difference was noted in the radial inclination, radial height, volar tilt, or ulnar variance between the 2 groups (Table 3).

**Table 3:** Comparison of radiologic outcomes between 2 groups.

Variables	Conventional Henry approach			Trans-FCR approach		
	3 mo	6 mo	1 yr	3 mo	6 mo	1 yr
Radial inclination (mm)	20.50 (17.25–25.00)	20.50 (17.25–25.00)	20.50 (17.25–25.00)	21.00 (20.00–22.00)	21.00 (20.00–22.00)	21.00 (20.00–22.00)
Radial height (mm)	10.30 (10.25–12.00)	10.30 (10.25–12.00)	10.30 (10.25–12.00)	10.50 (10.25–12.75)	10.50 (10.25–12.75)	10.50 (10.25–12.75)
Volar tilt (°)	15.00 (11.50–20.00)	15.00 (11.50–20.00)	15.00 (11.50–20.00)	16.50 (10.25–21.00)	16.50 (10.25–21.00)	16.50 (10.25–21.00)
Ulnar variance (mm)	0.47 (−0.75 to 2.45)	0.47 (−0.75 to 2.45)	0.47 (−0.75 to 2.45)	1.30 (−0.37 to 1.87)	1.30 (−0.37 to 1.87)	1.30 (−0.37 to 1.87)

Values in data cells represent median (interquartile range) or number.

FCR = flexor carpi radialis.

## 4. Discussion

In the current study, we compared the clinical and radiologic outcomes of the conventional Henry approach and trans-FCR approach for the treatment of distal radius fractures. We demonstrated that although both approaches yielded reliable results, the trans-FCR approach can cause discomfort such as tenderness to the area of the FCR tendon.

Various surgical approaches have been adopted for distal radius fractures, including the volar approach, radial approach, dorsal approach, and arthroscopic approach. With the growing advancement of fixed-angle volar plating, the volar approach has become increasingly popular in the surgical treatment of distal radius fractures.

Among the volar approaches to the distal radius, the conventional Henry and trans-FCR approaches provide excellent exposure of the volar surface of the distal radius and are commonly used for the surgical treatment of distal radius fractures.^[9,14–17]^ These 2 approaches can be accomplished with different superficial intervals. The conventional Henry approach utilizes the interval between the FCR tendon and the radial artery. Although careful attention is required to dissect the radial artery without causing damage, this approach does not require the opening the tendon sheath.^[11]^ On the other hand, the trans-FCR approach involves an incision of the FCR tendon sheath.^[3,18]^ Technically, the superficial layer of the sheath is incised longitudinally, and the FCR tendon is retracted medially, protecting the median nerve. The floor of the sheath is then incised to gain deep access. The trans-FCR approach has the advantages that direct radial artery dissection and isolation are not required. Additionally, the potential risk of iatrogenic injury to the palmar cutaneous branch of the median nerve can be prevented by avoiding ulnar dissection to the FCR tendon. Although these 2 approaches are typically used and showed reliable outcomes, there is a preference for each surgeon, and there are few studies comparing the clinical results of 2 approaches for distal radius fractures.

In this study, VAS score showed significant difference between the 2 groups at 6 months and 1 year after surgery. Nonetheless, since the median value of VAS score was 3 points or less, patients complained of discomfort that did not require further intervention. Actually, even if the VAS score itself had been improved, there were some patients whose tenderness persisted. Rather than pain, it is thought to be the discomfort such as tenderness shown in our study. Although tenderness was not lead to functional impairment of the wrist, these sensations were the cause of occasional complaints of discomfort.

We did not find a significant differences between the 2 groups regarding MMWS, ROM, grip strength, complications as well as radiologic parameters. Although it may be because of the relatively similar values among patients as well as small number of participants enrolled in the current study, we believe that the trans-FCR approach does not induce an additional risk of morbidity compared to the conventional Henry approach.

**Figure 4.**
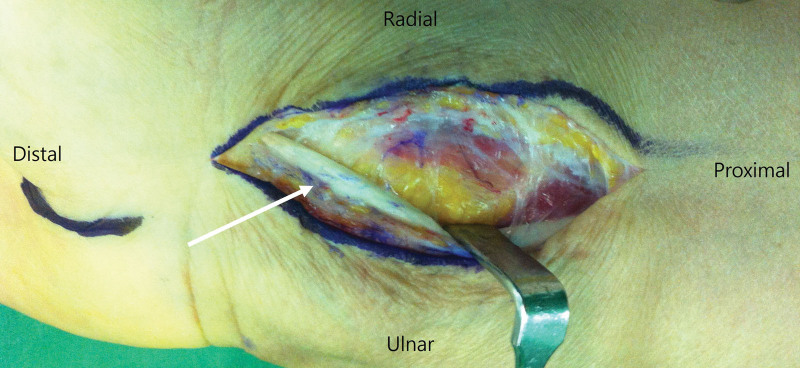
Intraoperative view of the adhesion around the flexor carpi radialis tendon during hardware removal in the case of the trans-flexor carpi radialis approach (white arrow).

Unlike previous studies, we evaluated the tenderness of the FPL tendon. We found that the trans-FCR approach can induce complaints of postoperative tenderness in the area of the FCR tendon. Basically, the FPL tendon is surrounded by the sheath and if the sheath is injured, scarring and peritendinous adhesions can occur through the healing mechanism.^[19,20]^ Therefore, we considered that the incised sheath of the FPL tendon during the trans-FCR approach is thickened by scar tissue, contributing to the tenderness felt along the FPL tendon. We performed hardware removal in all patients about 1 year postoperatively. There was a difference between the intraoperative findings of patient who underwent surgery through the conventional Henry approach and those who underwent surgery through the trans-FCR approach (Fig. 4). Although the generalizability of this finding is limited, the adhesions and fibrosis in the previous surgical field were more severe in patients who underwent surgery through the trans-FCR approach than in those treated with the conventional Henry approach. Therefore, we suggest that the trans-FCR approach may affect tendon sheath healing, leaving scarring of the surrounding tissues in the surgical field, which may result in discomfort such as tenderness. Currently, postoperative satisfaction has become a growing focus of surgical outcome evaluation and is considered important component of the movement toward patient-centered care. Although tenderness in the area of the FCR tendon does not cause functional impairment or serious problems of wrist joint, a small discomfort can affect the patient satisfaction after surgery. Therefore, the authors recommend that it may be helpful to consider these aspects when performing surgery for the treatment of distal radius fractures.

There are several limitations to this study. First, this study included a retrospective cohort, short-term follow-up, and relatively small sample size. Second, the process of healing might change depending on individual patient properties or fracture type; healing processes, such as inflammatory reactions, can alter tissue healing, which may affect the development of adhesions, fibrosis, or scarring.

## 5. Conclusion

The conventional Henry approach may show a faster recovery in terms of tenderness compared to the trans-FCR approach, but there is no significant difference in the final clinical and radiologic outcomes.

## Author contributions

**Conceptualization:** Do-Young Kim, Sang-Soo Lee, Jung-Taek Hwang.

**Data curation:** Bum-Jin Shim, Do-Young Kim, Min-Soo Cho.

**Formal analysis:** Bum-Jin Shim, Min-Soo Cho.

**Investigation:** Do-Young Kim, Sang-Soo Lee, Min-Soo Cho.

**Methodology:** Sang-Soo Lee.

**Supervision:** Jung-Taek Hwang.

**Validation:** Sang-Soo Lee, Jung-Taek Hwang.

**Visualization:** Jung-Taek Hwang.

**Writing – original draft:** Bum-Jin Shim.

**Writing – review & editing:** Bum-Jin Shim, Jung-Taek Hwang.
